# Nafithromycin in CABP: promise and precaution

**DOI:** 10.1016/j.lansea.2025.100692

**Published:** 2025-11-11

**Authors:** Nitin Gupta, Adil Rashid Khan, Praveen Kumar Tirlangi

**Affiliations:** a Department of Infectious Diseases, Kasturba Medical College, Manipal, Manipal Academy of Higher Education, Manipal, 576104, India; b Department of Medicine, All India Institute of Medical Sciences, New Delhi, Ansari Nagar, 110029, India

We read with interest the recent phase III trial comparing nafithromycin with moxifloxacin for community-acquired bacterial pneumonia (CABP).^1^ The investigators are to be commended for conducting a large, multicentre, double-blind study of a novel oral macrolide. The evaluation of a short-course regimen against an established comparator adds important data to the field and highlights the potential role of new macrolides in CABP management.

Nonetheless, certain aspects of the trial merit cautious interpretation. The study's central premise that nafithromycin may overcome increasing macrolide resistance in *Streptococcus pneumoniae* remains difficult to ascertain, given the paucity of pneumococcal cases recruited (7/149 nafithromycin and 12/156 moxifloxacin). The pathogen profile was dominated by atypical organisms, particularly *Chlamydia pneumoniae*, which was defined serologically by a fourfold rise in IgG titres. While widely used in research, this approach has limited specificity in daily clinical practice and may overestimate true infection rates.^2^ Together, and with an already wide non-inferiority margin, these factors significantly undermine the credibility of the proposed conclusions; One, they reduce the ability of the trial to demonstrate nafithromycin's performance against macrolide-resistant pneumococcal disease, the scenario of greatest clinical concern, and for which the drug was launched in the first place; Second, by including the cases which might not represent true infections, one is more likely to claim the non-inferiority given the spurious boost in event rate.

The study design itself also carries some limitations. The use of moxifloxacin as a comparator, despite its effectiveness, is complicated by evolving safety considerations and its reduced prominence in guideline-based therapy. Combined with the relatively wide non-inferiority margin, the use of a subjective, less robust, and noisy primary endpoint (symptom improvement by Day 4) subjects the study to misinterpretation and potentially makes it more likely to claim non-inferiority. Finally, the population enrolled was largely at lower risk, which may not fully reflect the patient groups most vulnerable to severe CABP.

In summary, this important trial demonstrates the promise of nafithromycin as a short-course macrolide regimen but leaves key questions unanswered. Further studies with a higher burden of pneumococcal disease, more specific diagnostic methods, and endpoints that reflect clinically meaningful outcomes will be valuable to clarify the role of nafithromycin in the treatment of CABP.

## Contributors

PKT, ARK, and NG contributed equally to the conception, drafting, and critical revision of the manuscript.

## Declaration of interests

The authors have nothing to declare.
